# New building blocks or dendritic pseudopeptides for metal chelating

**DOI:** 10.1186/s40064-016-1703-x

**Published:** 2016-01-20

**Authors:** Min Ruan, Irène Nicolas, Michèle Baudy-Floc’h

**Affiliations:** UMR CNRS 6226, Institut des Sciences Chimiques de Rennes (ISCR), 263 Avenue du Général Leclerc, 35042 Rennes Cedex, France

**Keywords:** Aza-β^3^-amino acids, Dendritic pseudopeptides, Aza-β^3^-peptides, Aza-NTA

## Abstract

Dendritic oligopeptides have been reported as useful building blocks for many interactions. Starting from hydrazine, we described an approach to create new dendritic pseudopeptides linked with biological systems, such as cell membrane, as chelate metal, Ni^2+^-nitrilotriacetic acid moieties which could target histidine rich peptides or proteins. Depending on the nature of these new chemical recognition units, they could be integrated into a peptide by coupling in *C* or *N*-termini.

**Graphical abstract:**
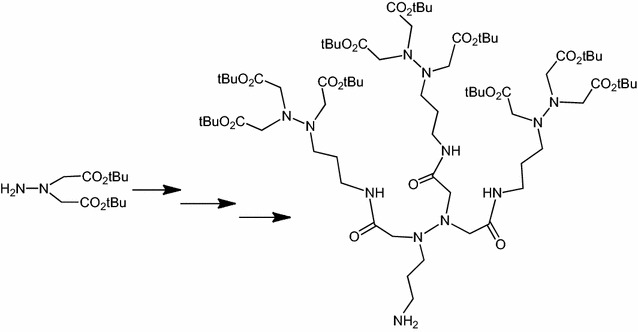
Dendrimer formation

Dendrimer formation

## Background

Unnatural amino acids constitute attractive targets for drug design. Disposing of a wide variety of unnatural amino acids allows the modulation of physical and chemical properties of the resulting peptide depending on the selected side chains (Gentilucci et al. 2010). The aza-β^3^-amino acids represent an exciting type of analogs of β^3^-amino acids in which the CH_β_ is replaced by a nitrogen stereocenter conferring a better flexibility to the pseudopeptide due to the side chain borne on a chiral nitrogen atom with non-fixed configuration (Busnel et al. 2005). Moreover, the backbone modification makes these molecules more stable towards proteolytic degradation (Dali et al. 2007; Laurencin et al. 2012).

Transition metals chelated by nitrilotriacetic acid (NTA) have been successfully applied for purification (Hochuli et al. 1987; Ueda et al. 2003) and detection of oligohistidine-tagged proteins (Hart et al. 2003; Lata et al. 2005), as well as for immobilization on surfaces (Sigal et al. 1996; Gershon and Khilko 1995; Schmid et al. 1997; Xu et al. 2004; Schmitt et al. 2000). The hexahistidine tag provides binding sites for three NTA moieties, indeed, multiple NTA moieties into single entities increase the affinity adaptors for oligohistidine-tagged proteins (Lata et al. 2005).

Herein we aimed to design new amino acid analogues or building blocks that can be incorporated into any polypeptide by solid-phase peptide synthesis. Potential applications of these metal-chelating units will be as metal sensors for synthetic receptors that interact specifically with histidine-tagged peptides.

## Results and discussion

**Scheme 1:**
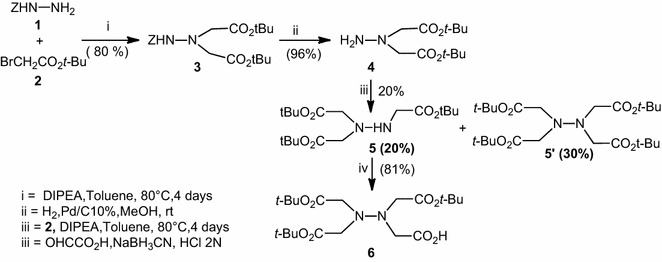
Synthesis of aza-NTA **6**

As part of our research program we develop new peptide analogues with potentially useful biological properties. For this purpose, we have developed synthetic strategy for aza-β^3^-aspartic acid (Busnel and Baudy-Floc’h 2007; Abbour and Baudy-Floc’h 2013). We observed that during this process a double substitution of benzyl carbazate **1** occurred to afford Z-aza-β^3^-Asp(O*t*-Bu)-O*t*-Bu **4** in 19 % yield. By using *tert*-butyl bromoacetate (3 eq) **2** and *N,N*-Diisopropyl ethylamine (DIPEA) (2 eq) **3** was obtained in 80 % yield (Scheme 1). The hydrogenolysis of **3** over 10 % Pd/C gave our precursor **4**. A nucleophilic substitution of **4** by *tert*-butyl bromoacetate (1 eq) in the presence of *N,N*-Diisopropyl ethylamine (DIPEA) (1 eq) afforded the expected building block **5** with one azanitrilotriacetic acid which could be coupled in *C*-termini (Scheme 1) with 20 % yield, we observed the formation of a secondary product **5**′. To increase the yield of compound **5**, we tried different solvents and different bases. The yield of **5** with acetonitrile/DIPEA or NEt_3_ was 18 %, with Toluene/potassium carbonate K_2_CO_3_ in suspension 20 %, and with μWaves (150 W, 90 °C, 45 min) 5 %.

Reductive amination of trisubstituted hydrazine **5** with glyoxylic acid in the presence of NaBH_3_CN led to the tetrasubstituted hydrazine **6** as new building block with one aza-NTA, which could be coupled in N-termini.

**Scheme 2:**
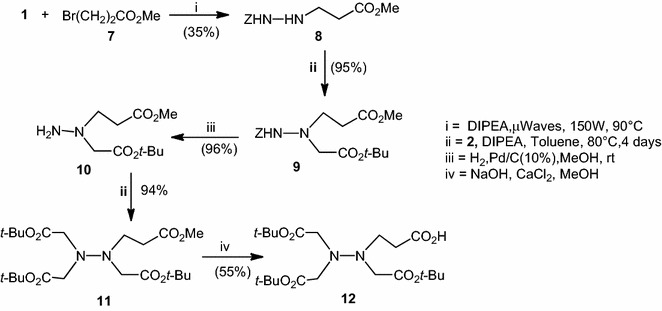
Synthesis of aza-NTA **12**

To create more flexibility to the aza-NTA, we first prepared the substituted aza-β^3^-glutamic ester **9**. Compound **8** was obtain by nucleophilic substitution of methyl 3-bromopropanoate **7** and benzyl carbazate **1** in the presence of DIPEA with only 17 % yield. The same reaction without solvent realized under microwaves activation provided **8** with 35 % yield. Then a second nucleophilic substitution of *tert*-butyl bromoacetate **2** with compound **8** and DIPEA led to Z-aza-β^3^Glu(OMe)-O*t*-Bu **9** with 96 % yield after stirring at 80 °C for 5 days. Then hydrogenolysis of **9** over 10 % Pd/C gave the monomer H-aza-β^3^Glu(OMe)-O*t*-Bu **10**. Nucleophilic substitution with two equivalents of *tert*-butyl bromoacetate **2**, H-aza-β^3^Glu(OMe)-O*t*-Bu **10** and DIPEA gave **11** (94 % yield). Methyl ester of **11** could be saponified (Pascal and Sol 1998) by sodium hydroxide in MeOH in the presence of CaCl_2_ affording the expected aza-NTA **12**, which could be coupled in N-termini of a peptide (Scheme 2).

**Scheme 3:**
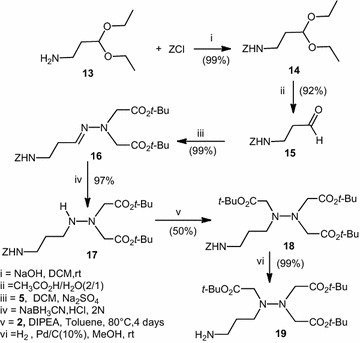
Synthesis of aza-NTA **19**

To obtain a new ligand with an amine function, which could be coupled on C-termini peptide we choose to work on ornithine analogue. The 1-amino-3,3-diethoxypropane precursor **13** was first *N*-protected with a benzyl group by reaction with benzylchloroformate under the presence of sodium hydroxide to afford benzyl 3,3-diethoxypropylcarbamate **14** with excellent yield (99 %). The acetal **14** was then treated with acetic acid and water (2/1) to give benzyl 2-formylethylcarbamate **15**. The condensation of **15** with our precursor **4** led to the hydrazone **16**. Reduction with sodium cyanoborohydride (NaBH_3_CN) gave the hydrazine **17**. Nucleophilic substitution of *tert*-butyl bromoacetate by hydrazine **17** afforded substituted aza-NTA **18**. Hydrogenolysis of **18** under 10 % Pd/C, gave a new ligand aza-NTA **19**, bearing a long amino chain with more flexibility (Scheme 3).

**Scheme 4:**
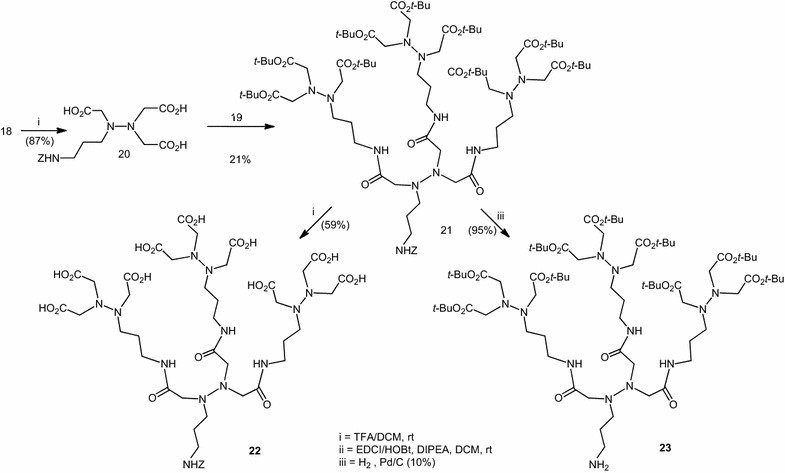
Synthesis of multimeric aza-NTA or dendritic pseudopeptides

Our goal was to get multimeric aza-NTA in order to increase the affinity to histidine tag proteins. Thus we built the dendritic pseudopeptides starting from our two building blocks **18** and **19**. Deprotection of acid functions of **18** with TFA afforded **20**. Then dendritic pseudopeptides or Z-aza-tris-NTA-*t*Bu **21** were synthesized via standard EDCI coupling of one equivalent of the *C*-deprotected intermediate **18** with three equivalent of the *N*-deprotected one **19**. We showed that it is possible to deprotect **21** either on *C*-ter to give Z-aza-tris-NTA-OH **22**, or on *N*-ter to lead to H-aza tris-NTA-*t*Bu **23**. NMR and HMRS mass spectrometry were used to verify the structure and purity of the amphiphilic dendritic peptides (Scheme 4).

## Conclusion

In summary, depending on the nature of our new chemical recognition units, these could be introduced by coupling in a peptide in C or N-termini as well as on peptidic chain. These new Ψ-NTA could open new ways to control protein–protein interactions, to design peptide-based interaction pairs or to generate switchable protein functions. Moreover it would be interesting to look at the self-assembly of our new dendric pseudopeptides.

## Methods

^1^H and ^13^C NMR spectra were recorded at 200 or 300 MHz and 75.5 MHz. ^1^H chemical shifts are reported in δ values in ppm relative to C*H*Cl_3_ (7.24 ppm) as internal standard and ^13^C chemical shifts are reported in ppm relative to C*D*Cl_3_ (77.0 ppm). Multiplicities in ^1^H NMR are reported as (br) broad, (s) singlet, (d) doublet, (t) triplet, (q) quartet, and (m) multiplet. The analytical laboratory from the Centre Régional de Mesures Physiques de l’Ouest performed electrospray mass spectrometry (HRMS, ESI) studies using MS/MS Mass spectrometer ZAB Spec TOF. Thin layer chromatography was performed on silica gel 60 F_254_ plates (Merck). Flash chromatography was performed on SP silica gel 60 (230–600) mesh ASTM. DCM was distilled from CaH_2_ under nitrogen.

### Nucleophilic substitution procedure

A mixture of hydrazine (4 mmol), DIPEA (1.1 g, 8 mmol) and *tert*-butyl bromoacetate **2** (1.87 g, 12 mmol) in toluene (20 mL) was stirred at 80 °C for 4 days. The solid was filtered and the filtrate was evaporated. The residue was purified by flash column chromatography on silica gel with DCM/EtOAc (9/1).

Compound **3**.

Yield: 88 %.

^1^H NMR (200 MHz, CDCl_3_): δ = 1.49 (s, 18H, *t*-Bu), 3.73 (s, 4H, N-*CH*_*2*_), 5.15 (s, 2H, CH_2_), 7.31 (m, 5H, C_6_H_5_).

^13^C NMR (75 MHz, CDCl_3_): δ = 28.1, 53.3, 66.9, 81.7, 128.1, 128.2, 128.5, 136.1, 156.8, 170.6.

HRMS (ESI): *m/z* [M +Na]^+^ calcd for C_20_H_30_N_2_O_6_Na: 417.2002; found 417.2002.

Compound **5**.

Yield: 20 %.

^1^H NMR (200 MHz, CDCl_3_): δ = 1.49 (s, 27H, *t*-Bu), 3.61 (s, 4H, N-*CH*_*2*_), 3.63 (s, 2H, N-*CH*_*2*_).

^13^C NMR (75 MHz, CDCl_3_): δ = 27.5, 56.0, 62.5, 63.5, 80.2, 173.9.

HRMS (ESI): *m/z* [M + H]^+^ calcd for C_18_H_35_N_2_O_6_: 375.2495; found 375.2495.

Compound Z-Aza-β^3^Glu(OtBu)-OMe **9**.

Yield: 94 %.

^1^H NMR (200 MHz, CDCl_3_): δ = 1.67 (s, 9H, *t*-Bu), 2.54 (m, 2H, CH_2_), 3.22 (m, 2H, N-*CH*_*2*_), 3.62 (m, 5H, CH_3_ + N-*CH*_*2*_), 5.12 (s, 2H, CH_2_), 7.40 (m, 5H, C_6_H_5_).

^13^C NMR (75 MHz, CDCl_3_): δ = 26.6, 31.2, 41.7, 48.6, 60.3, 66.4, 128.6, 128.7, 128.8, 128.9, 129.0, 172.4, 173.4, 173.8.

HRMS (ESI): *m/z* [M + H]^+^ calcd for C_18_H_27_N_2_O_6_: 367.18691; found 367.1898.

HRMS (ESI): *m/z* [M + Na]^+^ calcd for C_18_H_26_N_2_O_6_Na: 389.16886; found 389.1694.

Compound **11**.

Yield: 99 %.

^1^H NMR (200 MHz, CDCl_3_): δ = 1.42 (s, 9H, *t*-Bu), 2.47 (m, 2H, CH_2_), 3.01 (m, 2H, N-*CH*_*2*_), 3.41 (s, 4H, N-*CH*_*2*_), 3.51 (s, 2H, N-*CH*_*2*_), 3.64 (s, 3H, CH_3_).

^13^C NMR (75 MHz, CDCl_3_): δ = 28.6, 33.2, 52.1, 52.4, 57.4, 80.1, 169.6, 173.2.

HRMS (ESI): *m/z* [M + H]^+^ calcd for C_22_H_41_N_2_O_8_: 461.2863; found 461.2856.

Compound **18**.

Yield: 50 %.

^1^H NMR (200 MHz, CDCl_3_): δ = 1.47 (s br, 27H, *t*-Bu), 1.77 (m, 2H, CH_2_), 2.75 (m, 2H, CH_2_), 3.38 (m, 2H, N-*CH*_*2*_), 3.48 (s, 2H, N-*CH*_*2*_), 3.61 (s, 4H, N-*CH*_*2*_), 5.15 (s, 2H, CH_2_), 7.31 (m, 5H, C_6_H_5_).

^13^C NMR (75 MHz, CDCl_3_): δ = 24.2, 28.6, 33.2, 52.1, 56.4, 57.4, 66.7, 80.1, 127.2, 127.5, 128.4, 135.8, 157.8, 169.6.

HRMS (ESI): *m/z* [M + H]^+^ calcd for C_29_H_48_N_3_O_8_: 566.3441; found 566. 3221.

Compound **8**: A mixture of Z-carbazate **1** (2 g, 12 mmol), methyl 3- bromopropanoate **7** (2 g, 12 mmol), DIPEA (1.56 g, 12 mmol), NaI (1.2 g, 12 mmol) in toluene (20 mL) was stirred at 80 °C for 7 days. The solid was filtered and the filtrate was evaporated under reduced pressure. The residue was purified by column chromatography on silica gel with DCM/EtOAc (9/1) to afford **8**.

Yield: 0.5 g (17 %).

The same reaction was realized without solvent by microwave activation (SYNTHEWAVE 402: 150 W, 45 min, 90 °C) to get **8**.

Yield: 1.1 g (35 %).

1H NMR (200 MHz, CDCl_3_): δ = 2.55 (t, 2H, CH_2_), 3.21(t, 2H, N-*CH*_*2*_), 3.72(s, 3H, CH_3_), 5.19 (s, 2H, CH_2_), 7.40 (s, 5H, C_6_H_5_).

^13^C NMR (75 MHz, CDCl_3_): δ = 31.2, 38.8, 41.5, 47.8, 128.6, 128.7, 128.8 128.9, 129.0, 134.6, 172.5, 173.9.

HRMS (ESI): *m/z* [M + H]^+^ calcd for C_12_H_16_N_2_O_4_: 252.1110; found 252.1111.

Compound aza-NTA **6**.

To a solution of substituted hydrazine **5** (1.9 g, 5 mmol) in DCM/MeOH (10/25 mL), glyoxylic acid monohydrate (0.44 g, 1.2 equiv) was added. Then NaBH_3_CN (0.46 g, 1.5 eq) was added fractionally into the above mixture, which was maintained under stirring for 1 h, and the pH was maintain at 3 by addition of 2 N HCl. Then HCl was added until pH 1 over 10 min and finally increased to 4-5 with a saturated NaHCO_3_ solution. The mixture was filtered, concentrated, taken up with EtOAc (10 mL) and washed with 2 N HCl solution and brine. The organic layer was dried over anhydrous Na_2_SO_4_ and concentrated to give a crude foam, which was triturated in Et_2_O to give **6**, which was purified by chromatography on silica gel (DCM/MeOH: 9/1).

Yield: 1.8 g (81 %).

^1^H NMR (200 MHz, CDCl_3_): δ = 1.50 (s, 27H, *t*-Bu), 3.64 (s, 2H, N-*CH*_*2*_), 3.66 (s, 6H, N-*CH*_*2*_).

^13^C NMR (75 MHz, CDCl_3_): δ = 26.5, 56.8, 61.0, 63.5, 63.9, 79.8, 174.9, 180.9.

HRMS (ESI): *m/z* [M + H]^+^ calcd for C_20_H_37_N_2_O_8_: 433.25499; found 433.256.

Compound Aza NTA **12.**

**11** (1.2 g, 6 mmol) was dissolved in MeOH (14 mL) and CaCl_2_ (2.6 g, 0.4 M), NaOH (0.125 g, 3.1 mmol) was dissolved in H_2_O (6 mL). These two solutions were mixed and stirred at room temperature for 6 h. Then, 2 N HCl solution was added to get a neutral pH. Evaporation of methanol under vacuum and extraction with EtOAc (20 mL × 2) led to an organic phase, which was washed with 2 N HCl solution (20 mL) and brine (20 mL). The solvent was evaporated under vacuum and the residue was purified by column chromatography on silica gel with DCM/EtOAc (8/1) to afford the triester **12**.

Yield: 0.65 g (55 %).

^1^H NMR (200 MHz, CDCl_3_): δ = 1.53 (s, 27H, *t*-Bu), 2.55 (m, 2H, CH_2_), 3.11 (m, 2H, N-*CH*_*2*_), 3.57 (s, 4H, N-*CH*_*2*_), 3.62 (s, 2H, CH_2_, N-*CH*_*2*_).

^13^C NMR (75 MHz, CDCl_3_): δ = 28.0, 28.1, 28.3, 33.6, 49.9, 51.5, 51.7, 53.7, 80.5, 80.9, 81.1, 163.6, 165.6, 167.1, 172.2.

HRMS (ESI): *m/z* [M + Na]^+^ calcd for C_21_H_38_N_2_O_8_Na: 469.25259; found 469.2489.

### Hydrogenolysis procedure

Hydrazine (18 mmol) was dissolved in MeOH (50 mL) and 10 % Pd/C (0.7 g) was added. The mixture was stirred under hydrogen atmosphere at room temperature for 6 h. The catalyst was eliminated by filtration through a Celite^®^ pad and the solvent removed under vacuum to obtain colorless product **4**, **10**, **19** and **23** enough pure.

Compound **4**.

Yield: 96 %.

^1^H NMR (200 MHz, CDCl_3_): δ = 1.51 (s, 18H, *t*-Bu), 3.15 (br, 2H, NH_2_), 3.66 (s, 4H, CH_2_).

^13^C NMR (75 MHz, CDCl_3_): δ = 27.6, 62.6, 79.9, 170.6.

HRMS (ESI): *m/z* [M + H]^+^ calcd for C_12_H_25_N_2_O_4_: 261.18143; found 261.1815.

Compound **10**.

Yield: 99 %.

^1^H NMR (200 MHz, CDCl_3_): δ = 1.47 (s, 9H, *t*-Bu), 2.74 (m, 2H, CH_2_), 3.24 (m, 2H, N-*CH*_*2*_), 3.50 (br, 2H, NH_2_), 3.62 (s, 3H, CH_3_), 4.25 (s, 2H, N-*CH*_*2*_).

^13^C NMR (75 MHz, CDCl_3_): δ = 26.9, 31.1, 50.3, 54.3, 65.3, 81.6, 169.4, 173.4.

HRMS (ESI): *m/z* [M + H]^+^ calcd for C_10_H_21_N_2_O_4_: 233.15013; found 233.1498.

Compound Aza NTA **19**.

Yield: 99 %.

^1^H NMR (200 MHz, CDCl_3_): δ = 1.50 (s br, 27H, *t*-Bu), 2.14 (m, 2H, CH_2_), 2.73 (m, 2H, N-*CH*_*2*_), 3.31(m, 2H, N-*CH*_*2*_), 3.40 (s, 2H, N-*CH*_*2*_), 3.48 (br, 2H, NH_2_), 3.53(s, 4H, N-*CH*_*2*_).

^13^C NMR (75 MHz, CDCl_3_): δ = 27.1, 27.9, 38.1, 50.3, 54.3, 55.3, 81.6, 169.4.

HRMS (ESI): *m/z* [M + H]^+^ calcd for C_21_H_42_N_3_O_6_: 432.30736; found 432.2978.

Compound **23**.

Yield: 95 %.

^1^H NMR (300 MHz, CDCl_3_): *δ* = 1.53 (br, 81H, *t*-Bu), 1.75 (m, 8H, CH_2_), 2.58-2.72 (m, 10H, N-*CH*_*2*_), 3.41-3.58 (m, 30H, N-*CH*_*2*_).

^13^C NMR (75 MHz, CDCl_3_): δ = 26.8, 27.2, 37.3, 39.1, 51.4, 52.3, 57.5, 58.3, 169.7, 170.4.

HRMS (ESI): *m/z* [M + H]^+^ calcd for C_72_H_135_N_12_O_21_: 1503.9865; found: 1503.9764 (1 ppm).

Compound **14**.

A solution of 1-Amino-3,3-diethoxypropane **13** (2 g, 13.6 mmol) was added into a solution of NaOH (0.55 g, 13.6 mmol) in water (20 mL) and cooled at 0 °C. The solution of benzylchloride (2.32 g, 13.6 mmol) in DCM (20 mL) was slowly added into the cooled solution. The mixture was stirred at room temperature for 12 h. After washing with H_2_O, the organic phase was dried and concentrated under vacuum to give benzyl 3,3-diethoxy propyl carbamate **14**.

Yield: 3.9 g (99 %).

^1^H NMR (200 MHz, CDCl_3_): δ = 1.24 (t, 6H, *J* = 7 Hz, OCH_2_*CH*_*3*_), 1.85 (m, 2H, CH_2_), 3.33 (m, 2H, CH_2_), 3.53 (m, 4H, O*CH*_*2*_CH_3_), 4.59 (t, 1H, *J* = 5.4 Hz, CH), 5.14 (s, 2H, CH_2_), 7.39 (m, 5H, C_6_H_5_).

^13^C NMR (75 MHz, CDCl_3_): δ = 16.5, 30.3, 32.8, 63.6, 66.9, 127.5, 127.7, 128.7, 136.5, 157.1.

HRMS (ESI): *m/z* [M + H]^+^ calcd for C_15_H_24_NO_4_: 282.1834; found 282.1836.

Compound **15**.

Benzyl 3, 3-diethoxypropyl carbamate **14** (3.9 g, 13.6 mmol) was dissolved into a solution of CH_3_CO_2_H/H_2_O (7 mL/3.5 mL), and stirred for 5 h. NaHCO_3_ was added into the solution until basic pH. The product was extracted with Et_2_O (20 mL × 2) and dried over Na_2_SO_4_. The solvent was removed under vacuum to afford benzyl (3-oxopropyl) carbamate **15**, which was used immediately without purification.

Yield: 2.6 g, (92 %).

^1^H NMR (200 MHz, CDCl_3_): δ = 2.78 (m, 2H, CH_2_), 3.53 (m, 2H, N-*CH*_*2*_), 5.13 (s, 2H, CH_2_), 7.39 (m, 5H, C_6_H_5_), 9.84 (m, 1H, C*H*O).

^13^C NMR (75 MHz, CDCl_3_): δ = 34.2, 40.8, 65.8, 127.6, 128.7, 128.8, 137.6, 152.5, 193.9.

Compound **16**.

Benzyl (3-oxopropyl) carbamate **15** (2.6 g, 12.6 mmol) and **5** (3.25 g, 12.6 mmol) were dissolved into DCM (30 mL), Na_2_SO_4_ was added to absorb the water and accelerated the reaction. The solution was stirred overnight at room temperature and filtrated to remove Na_2_SO_4_. The filtrate was concentrated and purified by chromatography over silica gel with PE/EtOAc (7/3) first and then (6/4) to give pure hydrazone **16**.

Yield: 5.63 g (99 %).

^1^H NMR (CDCl_3_): δ = 1.47 (s, 18H, *t*-Bu), 2.45 (m, 2H, CH_2_), 3.48 (m, 2H, CH_2_), 3.95 (s, 4H, N-*CH*_*2*_), 5.09 (s, 2H, CH_2_), 5.31(s, 1H, NH), 6.52 (t, 1H, *J* = 4.2 Hz, CH), 7.38 (m, 5H, C_6_H_5_).

^13^C NMR (CDCl_3_): δ = 28.0, 32.6, 38.1, 56.6, 66.6, 79.4, 127.9, 128.1, 128.4, 154.9, 173.6.

HRMS (ESI) *m/z* [M + H]^+^ calcd for C_23_H_36_N_3_O_6_: 450.2604; found 450.2559.

Compound **17**.

The hydrazone **16** (2.1 g, 4.68 mmol) was dissolved in MeOH (30 mL), NaBH_3_CN (0.35 g, 1.2 eq) was added by portions. 2 N HCl solution was used to maintain a pH 3 and then the mixture was stirred for 2 h. HCl 2 N was added until pH 1, and after 10 min, the pH was increased to 7-8 by adding NaHCO_3_. The solid was filtrated after 2 min, and the solvent was removed under vacuum and the crude product was dissolved into EtOAc (30 mL) and washed by H_2_O (2 × 20 mL). The organic phase was dried under Na_2_SO_4_ and the solvent was removed under vacuum to afford hydrazine **17**.

Yield: 2 g (97 %).

^1^H NMR (200 MHz, CDCl_3_): δ = 1.49 (s, 18H, *t*-Bu), 2.23 (m, 2H, CH_2_), 2.86 (m, 2H, CH_2_), 3.36 (m, 2H, N-*CH*_*2*_), 3.59 (s, 4H, N-*CH*_*2*_), 5.11 (s, 2H, CH_2_), 7.37 (m, 5H, C_6_H_5_).

^13^C NMR (75 MHz, CDCl_3_): δ = 25.9, 28.2, 36.6, 42.1, 56.6, 66.8, 81.4, 128.2, 128.4, 132.9, 158.9, 164.8.

HRMS (ESI): *m/z* [M + H]^+^ calcd. for C_23_H_38_N_3_O_6_: 452.2761; found 452.2754.

### Cleavage of *t*-Bu protection

2 mmol of protected compound were dissolved in the solution of DCM (5 mL)/TFA (5 mL), and stirred for 5 h. The solvent was removed under vacuum to get compounds **20** and **22**.

Compound **20**.

Yield: 87 %.

^1^H NMR (200 MHz, CDCl_3_): δ = 2.12 (m, 2H, CH_2_), 2.78 (m, 2H, N-*CH*_*2*_), 3.42 (m, 2H, N-*CH*_*2*_), 3.49 (s, 2H, N-*CH*_*2*_), 3.53(s, 4H, N-*CH*_*2*_), 4.88 (s, 2H, CH_2_), 7.11(m, 5H, C_6_H_5_).

^13^C NMR (75 MHz, CDCl_3_): δ = 24.4, 37.5, 51.2, 52.1, 57.9, 58.9, 66.8, 127.2, 127.6, 128.9, 134.9, 156.9, 172.8.

HRMS (ESI): *m/z* [M + H]^+^ calcd for C_17_H_24_N_3_O_8_: 398.1564; found 398.1498.

Compound **22**.

Yield: 59 %.

^1^H NMR (300 MHz, CDCl_3_): *δ* = 1.75 (m, 8H, CH_2_), 2.65 (m, 8H, N-*CH*_*2*_), 3.12-3.68 (m, 32H, N-*CH*_*2*_), 5.05 (s, 2H, CH_2_), 7.23 (m, 5H, C_6_H_5_).

^13^C NMR (75 MHz, CDCl_3_): δ = 24.9, 28.7, 37.6, 52.1, 52.3, 56.6, 58.6, 59.1, 56.3, 66.6, 127.1, 127.7, 128.9, 136.0, 155.9, 170.8, 171.4.

HRMS (ESI): *m/z* [M + H]^+^ calcd for C_44_H_68_N_12_O_23_: 1133.4599; found: 1133.4567 (1 ppm).

Compound Z-aza-NTA-*t*-Bu **21**.

A mixture of **18** (0.13 g, 0.30 mmol), **20** (0.43 g, 1 mmol), HOBt (0.18 g, 1.16 mmol), EDCI (0.23 g, 1.16 mmol), DIPEA (0.52 g, 4 mmol) in dry DCM (20 mL) was stirred at room temperature for 2 weeks. The solution was washed with 0.5 N HCl solution (10 mL), and then with H_2_O (20 mL), and brine (10 mL). The organic solution was dried over anhydrous Na_2_SO_4_ and evaporated under vacuum and purified by flash chromatography with DCM/EtOAc (9/1) to afford multimaric **21**.

Yield: 0.11 g (21 %).

^1^H NMR (300 MHz, CDCl_3_): *δ* = 1.45 (m, 81H, *t*-Bu), 1.77 (m, 8H, CH_2_), 2.75 (m, 8H, N-*CH*_*2*_), 3.12-3.68 (m, 32H, N-*CH*_*2*_), 5.09 (s, 2H, CH_2_), 7.33(m, 5H, C_6_H_5_).

^13^C NMR (75 MHz, CDCl_3_): 24.9, 28.7, 37.6, 52.1, 52.3, 56.6, 58.6, 59.1, 56.3, 66.6, 81.4, 127.2, 127.7, 128.6, 135.9, 156.9, 168.9, 169.8.

HRMS (ESI) *m/z* [M + H]^+^ calcd for C_80_H_141_N_12_O_23_: 1638.0233; found: 1638.0250 (1 ppm).

## Abbreviations

*t*-Bu: *tertio*-butyl
CHCl_3_: chloroform
DCM: dichloromethane
DIPEA: *N,N*-diisopropylethylamine
EDCI: 1-(3-dimethylaminopropyl)-3-ethylcarbodiimide
EtOAc: ethylacetate
Et_2_O: diethyl ether
HOBt: 1-hydroxy-benzotriazole
MeOH: methanol
MW: microwaves
NaBH_3_CN: sodium cyanoborohydride
NaOH: sodium hydroxide
Na_2_SO_4_: sodium sulfate
PE: petroleum ether
rt: room temperature
TEA: triethyl amine
TFA: trifluoro acetic acid
THF: tetrahydrofuran
Z: benzyloxycarbonyl
